# School-based peer-led diabetes intervention among female adolescents: a cluster randomized trial

**DOI:** 10.1186/s12889-023-15430-3

**Published:** 2023-06-17

**Authors:** Pooresmaeil Dorosteh Ameneh, Ghaffari Mohtasham, Rakhshanderou Sakineh, Mehrabi Yadollah, Ramezankhani Ali

**Affiliations:** 1 Ph. D Student of Health Education & Health Promotion, School of Public Health and Safety, Shahid Beheshti University of Medical Sciences, Tehran, Iran; 2 Professor of Health Education & Health Promotion, School of Public Health and Safety, Shahid Beheshti University of Medical Sciences, Tehran, Iran; 3 Assistant Professor of Health Education & Health Promotion, School of Public Health and Safety, Shahid Beheshti University of Medical Sciences, Tabnak Ave., Daneshjou Blvd., P.O, Velenjak, Tehran Iran; 4 Professor of Biostatistics, Department of Epidemiology, School of Public Health and Safety, Shahid Beheshti University of Medical Sciences, Tehran, Iran; 5 Professor of Health Education & Health Promotion School of Public Health and Safety, Shahid Beheshti University of Medical Sciences, Tehran, Iran

**Keywords:** Adolescents, Attitude, Behavior, Diabetes mellitus, Peer education, Knowledge

## Abstract

**Background:**

The prevalence of type 2 diabetes is increasing among adolescents and clear strategies are needed to prevent it. The aim of this study was to determine the effect of peer education on knowledge, health beliefs and preventive behaviors of type 2 diabetes in female adolescents.

**Methods:**

In this cluster randomized trial study, 168 students (84 people in each group) were enrolled. The data collection instrument was a questionnaire of knowledge (30 questions), health beliefs (16 questions) and behavior (20 questions) whose validity and reliability were confirmed. Then eight capable students were chosen as peer educators after being trained. The intervention group received 8 sessions of 90-min education through training, lectures, question and answer, and group discussion and with teaching aids such as pamphlets, educational clips and text messages. The post-test was administered two months after the treatment. Data collected using software SPSS16 and Chi-Square and ANCOVA test were used.

**Results:**

The result showed that the mean and standard deviation of general knowledge, disease symptoms, behavioral risk factors, mid-term outcomes and long-term outcomes, perceived self-efficacy, behavioral beliefs, perceived susceptibility, perceived severity, prevention of stress, healthy food/healthy diet, unhealthy food/unhealthy diet, high-risk behavior, and self-care in the intervention group has increased significantly 2 months after intervention compared of control group (*P* < 0.001).

**Conclusions:**

Peer education increased knowledge and improved adolescents' health beliefs and behaviors. Therefore, training in adolescence in order to prevention of diabetes can be considered as an effective step, and the use of peer-led education in this field is recommended.

**Trial registration:**

Trial registration number IRCT20200811048361N1 from School of Public Health & Neuroscience Research Center—Shahid Beheshti University of Medical Sciences. Date applied: 30/12/2020. Date assigned: 01/12/2020.

## Background

Diabetes is a chronic and metabolic disease with high levels of blood sugar that leads to several complications [1]. According to the International Diabetes Federation report in 2019, 463 million adults (20–79 years old) were suffering from type-2 diabetes. This figure is expected to rise to 783 million by 2045 [2]. In the Eastern Mediterranean region, Kuwait and Yemen recorded the highest (22%) and the lowest (3.9%) rate of type-2 diabetes respectively in 2019. Iran had an outbreak record of 9.4% [3]. Also, according to the World Health Organization report in 2016, the prevalence of diabetes in Iranian women at 11.1% and in men at 9.6% [4].

In addition, this serious disease is witnessed as a new clinical condition among children. There has been a surge in the number of type-2 diabetics among children and adolescents across all ethnic groups [5]. Reportedly, type-2 diabetes has been diagnosed on average among adolescents of 12–14 years of age, coinciding with the age of puberty [6]. In 2011, one percent of Iranian adolescents between 10 and 19 suffered from type-2 diabetes [7].

Inflicting harm to different parts of the body, Type-2 diabetes can increase the long-term possibility of death [8]. This disease can be more complicated and dangerous among adolescents compared to adults. Diagnosis of type-2 diabetes at younger ages contributes to more chronic cases of cardiovascular diseases, compared to those whose disease develop in middle age [9]. In addition, the diagnosis of type-2 diabetes among younger age groups has been concurrent with hypertension, hyperlipidemia, nephropathy, and retinopathy [10], which can cut back 15 years on their lives. In fact, the serious and chronic complications might arise by the age of 40 [11].

Multiple factors such as social, cultural, geographical, and environmental ones can contribute to type-2 diabetes [12]. The financial and health consequences of this pandemic call for urgent public reaction. Since medical costs are enormous, preventive measures must come into focus [13]. As a result, education and knowledge raising are so critical in preventing type-2 diabetes that ‘education and prevention’ was chosen as the slogan by the International Federation of Diabetes from 2009 to 2013 [14].

Health education programs can empower young people to change themselves by raising their knowledge, influencing their beliefs and health beliefs, and improving their decision-making skills [13]. Education plays a key role in adolescence, as this stage of life constitutes the basis for change and formation of healthy habits and behavior. In fact, these newly-formed habits often persist into adulthood. Meanwhile, girls and women are thought to hold a pre-eminent position compared to men since they are the recipients of much of health education and they are the ones who impart and implement this knowledge to leave a lasting impact on the future health of their family [13]. For optimum results, health interventions must be designed to suit cultural sensitivities and the age of adolescents while employing a wide variety of methods [14].

One of such methods is that of peer education. Peer-based health education entails a group of peers receiving health tips and lessons as well as acquiring relevant skills to teach their peers. The purpose of this method is not only to raise knowledge but also to help change health beliefs and behavior through peer education [15]. Relying on the five senses, this method can shape adolescents’ thinking and develop their creativity [16]. Several studies provide evidence that school is the best setting to access adolescents as it is the location where educators can intervene to change high-risk behavior among adolescents [17]. Considering the ever-increasing rate of diabetes and the fact that type-2 diabetes is more prevalent among women than men (both in Iran and in the world) according to World Health Organization [18], and a comprehensive interventional study, which investigated all the behavioral risk factors of type 2 diabetes in adolescents, was not found, so the current study aimed at determining the effect of peer education on knowledge, health beliefs and preventive behaviors of type 2 diabetes in female adolescents in Tehran.

## Materials & methods

### Dates periods of recruitment

Participants were enrolled from July 2021 to October 2021 and participated in the intervention from November 2021 to April 2022.

### Study design and sampling

In this cluster randomized trial study, was performed in Tehran. 168 eighth grade female students (84 students in the intervention group and 84 in the control group) based on their interest and informed consent and their Parents written informed consent participated in the study in 2021.

**Fig. 1:**
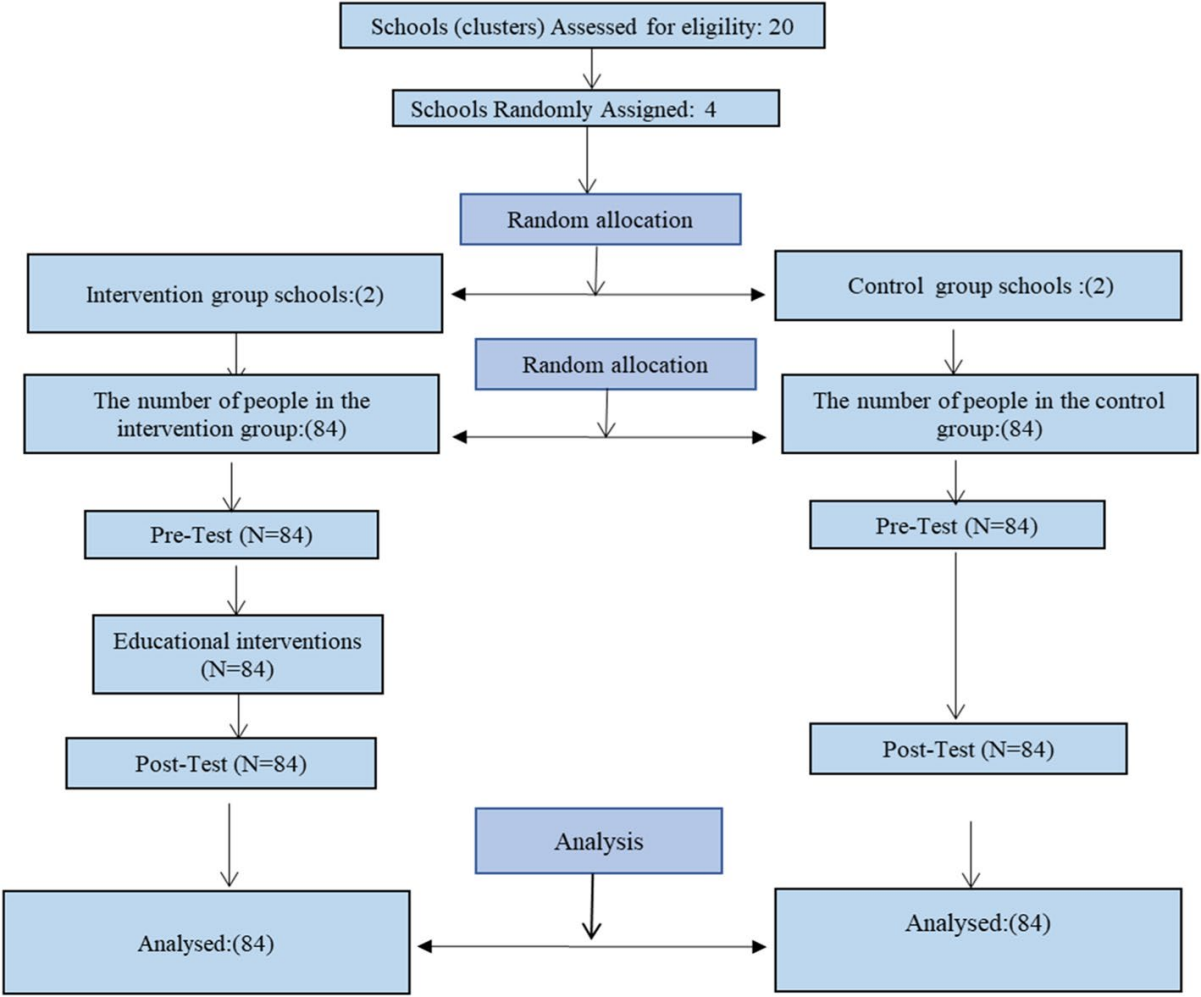
CONSORT Flow Diagram of Study

Initially, 14 district (a downtown area) in Tehran was selected out of the 22 districts by cluster randomly. Then four schools among the 20 girls' schools in this area, were chosen and allocated two groups of intervention and control randomly (Simple (. (2 schools for intervention groups and 2 schools for control groups) (Fig. 1). From each school 42 eligible students grade eight were selected by simple random sampling. It should be noted that the researcher generated a random allocation sequence and registered the participants and allocated them to two intervention and control groups (by simple random sampling).

### Sample size

Since, the standard deviation of the study variables was not known to the researcher, therefore based on the following formula, [19] $\frac{\Delta }{\text{S}}=$ 0.5(according to the researchers) and 10% sample drop, 84 sample calculated in each group:

$$\begin{array}{cccc}\mathrm{n}=\frac{{2\left({Z}_{1 -\frac{a}{2}}+{Z}_{1 -\beta }\right)}^{2}}{{\left(\frac{\Delta }{S}\right)}^{2}}& {Z}_{1 -\frac{a}{2}=1.96}& {Z}_{1 -\beta =1.28}& \frac{\Delta }{S} =0.5\end{array}$$

### Eligible criteria

#### Inclusion criteria

• Students’ willingness for participation
• Not suffering from type-1 or type-2 diabetes

#### Exclusion criteria

Reluctance to participate in the study at any stage.

### Data collection tools

Researcher-made questionnaire was used as data collection tool:

#### Designing the instrument:

- 1. Systematic review of literature and the relevant instruments: 38 questionnaires, 42 research papers, and 10 theses were consulted to design the questionnaire.
- 2. Determining and designing the items of the instrument through the existing documents, papers, and questionnaires in Iran and other countries: Relevant questions were extracted from various instruments and some questions were designed by virtue of papers and documents. Then, after negotiation with research team members the final items were added to the pool of questions and finally the first draft of the questionnaire with 108 questions was designed.
- 3. Validity of the instrument: Face validity, content validity, and construct validity were used to determine the validity of the instrument:

#### Reliability and validity

The tool’s validity was checked by face, content and construct validity. To determine face validity, the questionnaire was completed by 20 students. Content validity of the questionnaire was confirmed by qualitative and quantitative methods. In quantitative content analysis, Content Validity Ratio (CVR) and Content Validity Index (CVI) were evaluated by 11 experts. Then, questions with CVR of 0.59 and CVI of 0.79 and higher were preserved in the questionnaire. In order to evaluate the tool’s reliability, Cronbach’s alpha was calculated. Cronbach’s alpha of between 70 and 80% was set to be adequate and acceptable for internal consistency [20].

Test–retest was employed to investigate the stability of the instrument over time. The questionnaire was completed by 40 adolescents with an interval of 2 weeks. A correlation coefficient of higher than 0.7 was considered acceptable for internal correlation coefficient (ICC). The following formulas were used to evaluate the content validity ratio (CVR) and content validity index (CVI):

$$\begin{array}{cc}\mathrm{CVR} = \frac{{\mathrm{n}}_{\mathrm{E}} -{}^{\mathrm{N}}\!\left/ \!{}_{2}\right.}{{}^{\mathrm{N}}\!\left/ \!{}_{2}\right.}& \mathrm{CVI} = \sum \frac{\mathrm{Number of answer }3\mathrm{ or }4}{\mathrm{Total Number of answer}}\end{array}$$

Finally, the designed questionnaire had two parts:

Part one: Demographic questions about fathers’ job, mothers job, fathers’ education level, mothers’ education level and self-report economic situation.

Part two: Constructs of the knowledge (general knowledge, disease symptoms, behavioral risk factors, mid-term outcomes and long-term outcomes), health beliefs (perceived self-efficacy, behavioral beliefs, perceived susceptibility, and perceived severity) and behavior (prevention of stress, healthy food/healthy diet, unhealthy food/unhealthy diet, high-risk behavior, and self-care) (Table 1).

**Table 1:** Description of study instrument

Construct	**No. of Items (Format)**	**Scoring (Range)**
**Knowledge**	30 items (True – false—don’t know)	‘Correct’ response = 2, ‘don’t know’response = 1, ‘incorrect’ response = 0 (0–60)
1) General knowledge:) Regular physical activity can prevent type-2 diabetes. (	9 items (True – false—don’t know)	‘Correct’ response = 2, ‘don’t know’response = 1, ‘incorrect’ response = 0 (0–18)
2) Symptoms of disease (: Frequent urination is one of the symptoms of type-2 diabetes)	7 items (True – false—don’t know)	‘Correct’ response = 2, ‘don’t know’response = 1, ‘incorrect’ response = 0 (0–14)
3) Behavioral risk factors (: Smoking can increase type-2 diabetes risk)	4 items (True – false—don’t know)	‘Correct’ response = 2, ‘don’t know’response = 1, ‘incorrect’ response = 0 (0–8)
4) Medium-term consequences (: Type-2 diabetes can lead to hypertension)	4 items (True – false—don’t know)	‘Correct’ response = 2, ‘don’t know’response = 1, ‘incorrect’ response = 0 (0–8)
5) Long-term consequences (: Type-2 diabetes reduces the quality of life)	6 items (True – false—don’t know)	‘Correct’ response = 2, ‘don’t know’response = 1, ‘incorrect’ response = 0 (0–12)
**Health beliefs**	16 items/5-point Likert Scale (strongly disagree to strongly agree)	strongly disagree = 1, disagree = 2, no idea = 3, agree = 4, strongly agree = 5 (16–80)
1) Perceived Self-efficacy (: I can do regular exercise to prevent type-2 diabetes)	5 items/5-point Likert Scale (strongly disagree to strongly agree)	strongly disagree = 1, disagree = 2, no idea = 3, agree = 4, strongly agree = 5 (5–25)
2)Behavioral beliefs (: Depression and stress can contribute to type-2 diabetes)	5 items/5-point Likert Scale (strongly disagree to strongly agree)	strongly disagree = 1, disagree = 2, no idea = 3, agree = 4, strongly agree = 5 (5–25)
3)Severity Perceived (: Only overweight people contract diabetes)	4 items/5-point Likert Scale (strongly disagree to strongly agree)	strongly disagree = 1, disagree = 2, no idea = 3, agree = 4, strongly agree = 5 (4–20)
4)Perceived Sensitivity (: Diabetes can reduce life span)	2 items/5-point Likert Scale (strongly disagree to strongly agree)	strongly disagree = 1, disagree = 2, no idea = 3, agree = 4, strongly agree = 5 (2–10)
**Behavior**	20 items (Different options)	(Different options) (0–82)
1) Prevention of stress (: Regular exercise prevents stress and depression (	5 items (Always, Most of the time, Sometimes, Rarely, Never)	Always = 4, often = 3, sometimes = 2, rarely = 1, never = 0 (0–24)
2)Healthy Nutrition / Healthy Diet (: How much fruit do you eat every day? (One apple or one orange is equal to one unit. (	5 items (1–2 unit to I do not consume at all and 1 unit to I do not consume at all)	1–2 unit = 1, 2–3 unit = 2, 3–4 unit = 3, 5–4 unit = 4, I do not consume at all = 0 (0–8) 1 unit = 1, 2 units = 2, 3 units = 3, I do not consume at all = 0 (0–4)
3)Unhealthy Nutrition/ Unhealthy Diet (: How often do you eat fried food (fried chicken, fried fish, French fries, etc.)?	3 items (Every day of the week to I don't eat at all)	Every day of the week = 0, 5–6 times a week = 1, 3–4 times a week = 2, 1–2 times a week = 3, I don't eat at all = 4 (0–12)
4)High-risk Behaviors(:Do you smoke?(	3 items (Less than 1 h to I do not use TV, tablet, …at all and always to never)	Less than 1 h = 4, 1–2 h = 3, 2–3 h = 2, more than 3 h = 1, I do not use TV, tablet, mobile phone or electronic games at all = 5 (1–5) Always = 4, often = 3, sometimes = 2, rarely = 1, never = 0 (0–8)
5)Self-Care (: How often do you weigh yourself?)	3 items (Different options)	One vitamin D supplement a week = 4, one every 2 to 3 weeks = 3, one a month = 2, one a year = 1, I don't take it at all = 0 (0–4) Once a month = 4, once every 2–3 months = 3, once every 6 months = 2, once a year = 1, I don't measure at all = 0 (0–4) Less than 7 h = 2, 7–8 h = 3, more than 8 h = 1 (1–3)

### Intervention

The educational intervention was through peer education. Peer education has been employed as an effective strategy to combat diseases around the globe. Accordingly, people with shared characteristics such as age, gender, culture, education, and place of residence are assigned the task of imparting information with the aim of building knowledge and changing attitude and behavior among individuals, groups, and communities. Also, relying on all five senses, this method fosters thinking and creativity.

Based on recommended number for small groups^”^ size (8–12 individuals), research team decided to choose eight peers for education and communication with 84 students in intervention group.

Therefore, eight students known to be bright and keen on teaching were chosen as peer educators following suggestions from teachers and students themselves.

Training and briefing the peer educators was carried out by the researcher through a 2-day 2-h after-school workshop with lectures, questions and answers, and other educational tools. These sessions primarily focused on the purpose of education, communicating with students, implementing the intervention program, and the relevant type-2 diabetes discussions. Peers were evaluated by the researcher through pre-test and post-test in each training session. Then, the intervention process for the participants began. The pre-test was administered to both intervention and control groups to determine the educational needs with the aim of designing educational content. The results of statistical analysis helped to design the educational content. And educational content was extracted and prepared based on reliable sources. Educational and communicative strategies were used to implement intervention: eight sessions of 90 min each in a 45-day period. The peer educators taught the students by offering a definition of type-2 diabetes, as well as risk factors, symptoms, the complications of the disease, and the preventive measures. The intervention group received education through lectures, questions and answers, group discussions, pamphlets, messages, and educational video clips (motion graphics).

The control group received no treatment or education. Two months after the intervention, the questionnaire was completed again by the members of the control and intervention groups and the data was collected.

During the peer education, a school teacher and researcher were present to help maintain classroom order, attracting the cooperation and attention of students, provide support, trouble shooting, monitoring the work of peers and assess of adherence of peers to the protocol. Table 2 provides detailed information on session and duration, content and behavioral goals of education of the session, method of training and the evaluation method.

**Table 2:** Peer-led intervention activities for students

Construct	Session and duration	Content and Behavioral goals of education of the session	Method of training	Evaluation method
Knowledge	Session1 90 min	• Introduction to the program and Statement of the meeting's objectives • Definition of types of diabetes, type 2 diabetes • Understanding the symptoms of type 2 diabetes	Lectures Group discussion 4 Educational messages 1 Clip Pamphlet	Question and answer
	Session 2 90 min	• Understanding the risk factors for type 2 diabetes • Understanding the complications of type 2 diabetes	Group discussion 7 Educational messages 1Clip Pamphlet	Question and answer
Health beliefs	Session1 90 min	• Creating positive attitude towards complications of type 2 diabetes are permanent	Group discussion 2 Educational messages 1Clip Pamphlet	Question and answer
	Session 2 90 min	• Creating positive attitude towards preventability and curability of type 2 diabetes	Group discussion 8 Educational messages 1Clip Pamphlet	Question and answer
Behavior	Session1 90 min	• preventive implementations against type 2 diabetes	Group discussion 8 Educational messages 1Clip Pamphlet	Question and answer
	Session 2 90 min	• preventive implementations against type 2 diabetes	Group discussion 8 Educational messages 1Clip Pamphlet	Question and answer

### Data analysis

In this study Chi-square was used to check the homogeneity of samples as well as the consistency of contributing demographic factors in the research.

To check the normality of the data, Skewness and Kurtosis (In interval 2, -2) were calculated. Also, the ANCOVA was performed considering some of assumption, for example: The dependent variable and covariate were continues. Independent variable was consist of two categorical (experimental and control groups).There was no relationship between the observation in each group and between groups themselves. There was different participant in each group with no participant being in more than one group. There was homogeneity of variances (by using of Levine’s test) and there were no outliers among the research data.

Analysis of Covariance (ANCOVA) was utilized to compare the mean scores between the groups by adjusting for pretest effects. The level of statistical significance was set to be lower than 0.05. SPSS16 was used to analyze of collected data (pre-test) and to determine the effect of intervention 2 months after the educational intervention.

## Results

This study was conducted on 168 female students in the 14-year-olds age group. See the study steps in the CONSORT diagram (Fig. 1).

The participants' descriptive characteristics are listed in Table 3. Since some of socioeconomic variables affect people's knowledge, health beliefs, and behaviors related to type2 diabetes, it is necessary to consider these variables as covariates in intervention studies whit the aim of matching groups and adjusting the effects of these variables on the intervention.

**Table 3:** Demographic variables in intervention and control groups before the intervention

Variables	**Group Sub**	**Intervention group (*N* = 84) N (%**	**Control group (*N* = 84) N (%)**	***P* –value**
Fathers’ occupation	Employee	13(15.5)	19(22.6)	(*P* < 0.001)
	Self-employed	63(75)	17(20.2)	
	Unemployed	1 (1.2)	12(14.3)	
	Retired	2(2.4)	24(28.6)	
	Other	5(6)	12(14.3)	
Mothers’ occupation	Employed	15(17.9)	38(45.2)	(*P* < 0.001)
	House keeping	69(82.1)	46(54.8)	
Fathers’ education	Illiterate	2(2.3)	5(6)	*p* = 0.316
	Primary	5(6)	9(10.7)	
	Intermediate	21(25)	25(29.8)	
	Secondary	42(50)	37(44)	
	Institutes/College	14(16.7)	8(9.5)	
Mothers’ education	Illiterate	3(3.6)	7(8.3)	*p* = 0.059
	Primary	8(9.5)	17(20.2)	
	Intermediate	13(15.5)	18(21.4)	
	Secondary	43(51.2)	32(38.2)	
	Institutes/College	17(20.2)	10(11.9)	
Self-report Economic Situation	Poor	11(13.09)	12(14.3)	(*P* < 0.001)
	Middle	19(22.61)	21(25)	
	Good	42(50)	28(33.3)	
	Excellent	12(14.3)	23(27.4)	

In the intervention group, almost three quarters of students (75%) had fathers with their own business (freelancers). Most mothers (82.1%) were homemakers. 50% of students’ fathers and 51.2% of their mothers had high-school diploma. Most of the participants (50%) were reported to come from good -income families, with an exception of 14.3% who came from rich families.

In the control group, most of the students' fathers were retired (28.6%). Also, most mothers (54.8%) were homemakers. 44% of students’ fathers and 38.2% of their mothers had high-school diploma. In this group too, most of the participants (33.3%) were reported to come from good -income families, with an exception of 27.4% who came from rich families.

Despite randomization, the two groups differed slightly at baseline by some demographic characteristics. However, in the variables of age, gender, educational level and father's education, they were homogeneous. The socio demographic variables were measured by ordinal scale and cannot considered as a covariate. Since these socio demographic variables effect on Pretest, thus scores of each variable in pre-test considered as a covariate in ANCOVA. The result showed that the mean and standard deviation of all the components of knowledge, attitude and behavior in the intervention group has increased compared to the control group, and was significant (*P* < 0.001) The results of revealed that the intervention was successful in improving components of knowledge, attitude and behavior significantly in participants. (Table 4).

**Table 4:** Comparison of groups in terms of Knowledge, attitude and behavior before and after intervention

Variables	Groups	Before intervention Mean ± SD	2 months after intervention Mean ± SD	Mean Ddifference ± SD	*P*-value ANCOVA
Knowledge and its domains					
General Knowledge	Intervention	19.85 ± 79.23	12.84 ± 90.93	11.58 ± 0.42	*p* < 0.001
	Control	25.79 ± 57.60	25.72 ± 57.73		
Symptoms of Disease	Intervention	19.16 ± 65.98	14.33 ± 90.98	25.42 ± 0.36	*p* < 0.001
	Control	17.23 ± 54.76	17.28 ± 54.33		
Behavioral Risk factors	Intervention	20.42 ± 60.41	17.37 ± 76.78	17.11 ± 0.21	*p* < 0.001
	Control	29.13 ± 55.95	28.85 ± 55.20		
Medium-term Consequences	Intervention	23.51 ± 56.54	6.48 ± 98.09	41.67 ± 0.97	*p* < 0.001
	Control	11.03 ± 55.22	55.41 ± 22		
Long-term Consequences	Intervention	26.34 ± 65.95	5.56 ± 98.33	32.26 ± 1.06	*p* < 0.001
	Control	29.79 ± 57.73	29.53 ± 57.85		
Total Knowledge	Intervention	16.44 ± 67.63	7.56 ± 91.48	24.01 ± 0.53	*p* < 0.001
	Control	32.35 ± 56.17	17.43 ± 56.17		
Health beliefs and its domains					
Perceived Self-efficacy	Intervention	16.19 ± 71.38	11.31 ± 82.71	3.14 ± 0.44	*p* < 0.001
	Control	11.65 ± 77.76	12.25 ± 73.19		
Behavioral beliefs	Intervention	15.24 ± 66.38	11.23 ± 77.90	3.04 ± 0.45	*p* < 0.001
	Control	13.79 ± 74	12.01 ± 73.33		
Perceived Sensitivity	Intervention	14.65 ± 65.29	12.34 ± 76.25	1.43 ± 0.21	*p* < 0.001
	Control	16.24 ± 70.11	70.67 ± 15		
Severity Perceived	Intervention	19.51 ± 69.76	14.22 ± 80.23	11.19 ± 0.36	*p* < 0.001
	Control	20.90 ± 66.66	20.83 ± 65.95		
Total Health beliefs	Intervention	11.39 ± 68.09	8.02 ± 79.28	2.56 ± 0.36	*p* < 0.001
	Control	28.87 ± 73.8	53.76 ± 71.7		
Behavior and its domains					
prevention of Stress	Intervention	25.94 ± 62.89	19.37 ± 78.42	13.64 ± 0.53	*p* < 0.001
	Control	20.06 ± 64.13	20.14 ± 63.54		
Healthy Nutrition	Intervention	19.78 ± 45.96	18.16 ± 64.02	17.92 ± 0.15	*p* < 0.001
	Control	19.78 ± 45.96	15.51 ± 46.09		
Unhealthy Nutrition	Intervention	22.80 ± 50.39	16.41 ± 61.60	11.35 ± 0.6	*p* < 0.001
	Control	13.08 ± 63.88	12.78 ± 63.69		
High-risk Behaviors	Intervention	17.41 ± 74.08	7.01 ± 80.76	7.41 ± 0.65	*p* < 0.001
	Control	16.92 ± 62.36	16.99 ± 61.63		
Self-Care	Intervention	20.97 ± 65.15	14.83 ± 87.68	13.96 ± 0.45	*p* < 0.001
	Control	17.58 ± 59.41	17.87 ± 59.84		
Total Behavior	Intervention	15.15 ± 59.24	8.32 ± 81.31	7.71 ± 0.62	*p* < 0.001
	Control	7.83 ± 59	7.96 ± 73.36		

**Fig. 2:**
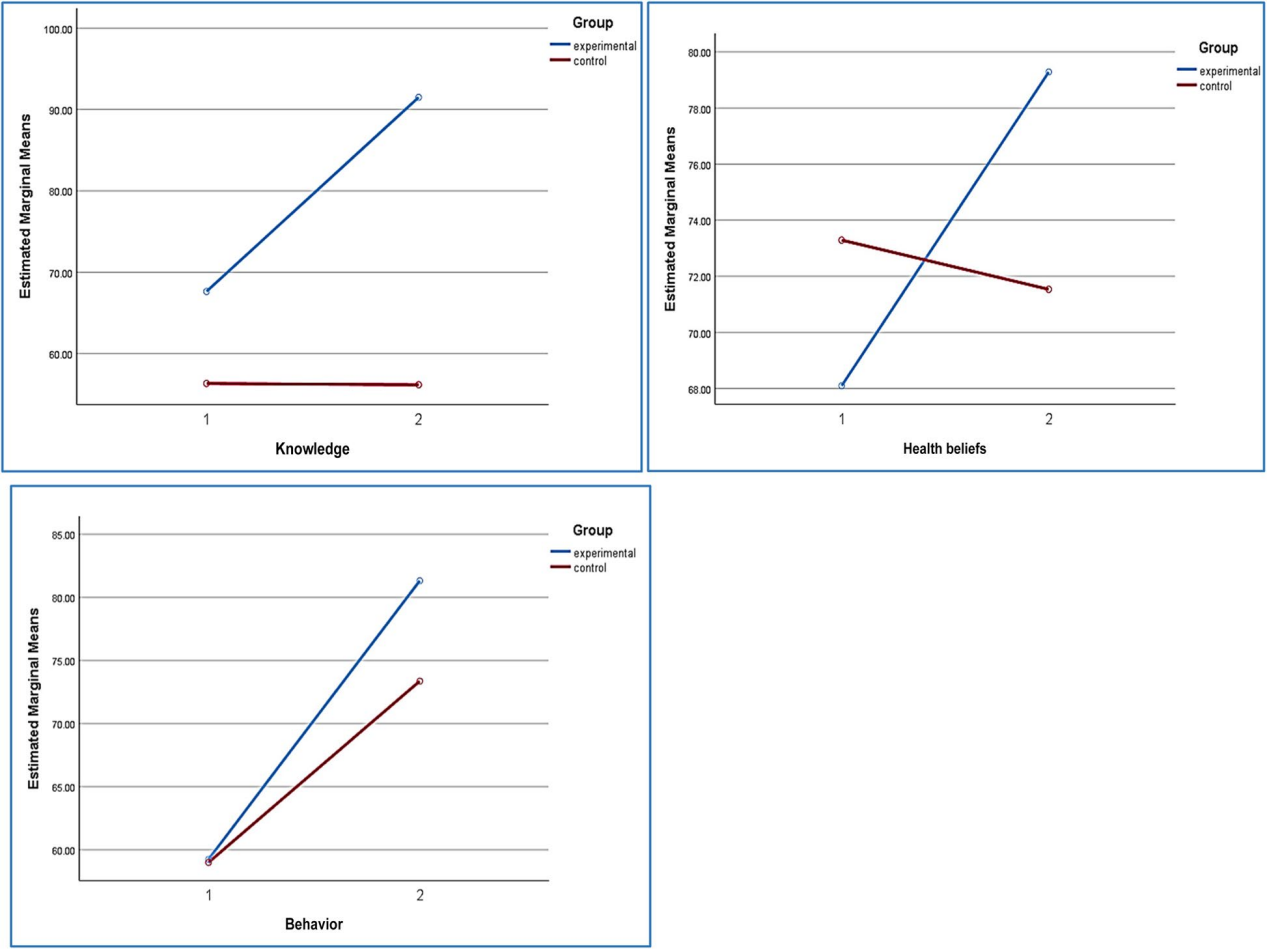
Estimated Marginal Means of knowledge, Health beliefs and Behavior

The mean score of knowledge, health beliefs and behavior in the experimental and control groups before and after the intervention is presented in Fig. 2. According to the figures, the mean of the variables in the intervention group has increased significantly 2 months after intervention compared of control group.

## Discussion

The main purpose of this research study was to determine the effect of peer education on knowledge, attitude, and preventive measures against type-2 diabetes among female adolescents. The results show that the peer educational intervention had positive effects on adolescents’ knowledge. Banerjeea et al. also witnessed significant differences regarding the knowledge of the definition, symptoms, and complications of type-2 diabetes after intervention among adolescents in the treatment group [21]. In a study aiming at investigating the relationship between preventive intervention and behavior-determining factors, Braver concluded that education could heighten knowledge among people prone to type-2 diabetes [22]. Safajoo et al. conducted a study based on health belief model and explored its effect on preventive measures against type-2 diabetes. They pointed out that education significantly increased the average score of knowledge among students [23]. Knowledge is, in fact, knowing what is required to change and modify behavior [24]. Increased knowledge in the intervention group is considered to be paramount because a knowledge of symptoms, risk factors, and the complications of type-2 diabetes is seen as an essential prerequisite to taking the right attitude as well as adopting the right approach toward type-2 diabetes.

Komolafe et al. believe that education is an effective tool to build knowledge and to effect behavioral changes to adolescents’ understanding of their state of health [25]. According to the results, peer education programs can raise knowledge of symptoms, risk factors, and the complications of type-2 diabetes. In addition, the results stress the necessity of such intervention programs for all students. Adolescents’ knowledge of type-2 diabetes can contribute to an early diagnosis of the disease and a reduction of its side effects. This ideal can be accomplished through gaining an adequate knowledge of the disease in the early stages of life. It seems that peer education can produce desired results in a shorter space of time compared to other methods. As the peer educator is selected from among the students in a class, it is conceivable that he/she can create a stronger impact on students in comparison with other methods of instruction [26].

Intervention led to a radical change of attitude toward type-2 diabetes in this study. Although the control group received no education, two months after the intervention there seemed to be perceptible differences, but this change was not statistically significant. One plausible explanation suggests that the control group has been influenced by the pre-test and they have gained some information about type-2 diabetes from other sources.

Peyman et al. conducted a 4-session educational intervention regarding diabetes that resulted in changing students’ attitude toward preventive measures adopted against type-2 diabetes [27]. Maleki et al. also concluded that implementing educational intervention positively influenced the female students’ attitude [28]. An individual’s attitude is one’s inclination to show an acquired positive or negative reaction to an object, situation, concept, or a certain person [29]. Positive or negative health beliefs can contribute to the development of chronic diseases [30] and adopting healthy behavior and measures might depend on the person’s attitude toward the risk factor, that is, the perceived sensitivity – one’s belief and perception of the potential risk – and the perceived severity – one’s perception of the side effects and complications of the disease [31].

Based on the results of this study, the average score of students saw an increase after peer education was implemented, indicating the positive effect of educational intervention. This increase, though to a lesser degree, was also witnessed in the control group. The findings regard intervention in the form of peer education as an efficient method to change adolescents’ behavior and encourage healthy habits and behavior. The present results are consistent with what Braver concluded in the Netherlands: education led to an increase in the consumption of healthy food such as fruit and fiber from bread, and to a reduction in consuming fat [22]. The findings also match those of Aguiar et al. who carried out an all-around 6-month intervention in the participants’ lifestyle. After the intervention, the participants were seen to consume healthier food [32]. Safajoo et al. also showed that educational intervention significantly increased the average scores in students’ behavior regarding type-2 diabetes [23] Behavior is a strong predictor of risk factor for type-2 diabetes, and it is essential for researchers to focus on methods of behavior change [33]. Behavior is, in fact, a collection of observable manners and actions adopted by an individual in response to a stimulus [34]. Early preventive measures against diabetes should target adolescents. Today, adolescents lead an unhealthy lifestyle and such unhealthy way of life can contribute to type-2 diabetes [35]. Following the results of the present study, peer education is believed to be an effective method of effecting changes to adolescents’ behavior and habits. Putting this method of education into practice can result in fundamental changes to adolescents’ behavior. As the inheritors of the future world, most vulnerable adolescents are at an impressionable age. Such interventions are the very steps necessary to take to promote a healthy lifestyle and community. Designing proper interventions can lead us to prevent and control this disease.

### Implications

A firm action to take in the prevention of type-2 diabetes is keeping adolescents under control in every place where they spend their time. School setting is considered crucial in promoting and implementing positive changes to adolescents’ habits School health strategies are a critical component of multidisciplinary and population approaches that are needed to combat the adolescent type 2 diabetes epidemic. The findings of this study demonstrated that intervention in the form of peer education in school setting raised adolescents’ knowledge, changed their attitude, and offered them practical knowledge of type-2 diabetes. Since peer-education intervention had never been implemented in school settings, this study can set an example and give an insight into the practicalities of peer education. It might as well provide the foundation for future studies to investigate the effectiveness of this approach, particularly, in cases where exploiting peer networks is an option.

### Limitations and strengths

The limitations of the study include impossibility of assessing the long-term effect of the intervention, individual’s responses to the questions. Another limitation of the study was self-report in response to behavioral questions (instead of objective observation), also since the questionnaire has been validated internally thus, the generalizability of the result is limited.

This study benefited from some strength including face-to-face education, use peers for education and having a control group and equal chance of being selected in the interventional or control groups in a cluster-randomized trial.

## Conclusion

The results of the study show that educational intervention of School-based within peer education framework can contribute to enhanced knowledge and Improve health beliefs and behaviors among female students. Applying this study to a similar population may reduce the risk of Type 2 diabetes in them.

Therefore, implementing healthcare policies in schools should be prioritized by authorities and public health experts. The results of this research can be used in Peer intervention strategies so as to effect health measures changes. Future studies are recommended to Inclusion of educational materials about prevention of diabetes in textbooks and enriching the school libraries with books about health issues. Also, the use of effective and useful method of peers in educational interventions to prevent of type 2 diabetes.

## Data Availability

The datasets used and analyzed during the current study are available from the corresponding author on reasonable request.

## Abbreviations

IDF: International diabetes federation
WHO: World health organization
